# A Preliminary Analysis of the Importance of Distance, Angle, and Insight When Soccer Referees Make Penalty Decisions

**DOI:** 10.3389/fspor.2020.595703

**Published:** 2021-01-08

**Authors:** Bjørn Tore Johansen, Martin Kjeøen Erikstad

**Affiliations:** Department of Sport Science and Physical Education, Faculty of Health and Sport Sciences, University of Agder, Kristiansand, Norway

**Keywords:** penalty situations, distance, angle, insight, decision-making skills

## Abstract

Soccer referees move freely on the pitch to place themselves in the best location for making decisions. While Football Association UK (FA) highlights that a referee should never be more than 20 m away from the playing situation, previous studies have been inconsistent in indicating appropriate distance to a situation for increasing the likelihood of a correct decision. Further, appropriate angle and insight are also likely to influence the correctness of referees' decisions. The aim of this study was to provide an initial investigation of elite referees' positioning in the field (distance, angle, and insight) when making correct and erroneous decisions in potential penalty situations. An expert panel (EP) consisting of two active referees with relevant academic background analyzed referees positioning when making correct or erroneous decisions regarding penalties. The EP were asked to qualitatively analyze referees positioning in selected video clips by using recommended technical refereeing criteria and practical guidelines (i.e., the referee's distance from, angle to, and insight into the penalty situations). Of the 42 situations evaluated, the results revealed that the EP termed the referees positioning as good in terms of angle and insight in 25 and 21 situations, respectively. The angle was average in seven situations and poor in 10 situations, and the insight were average in 10 situations and poor in 11 situations. The match referee was <10 meters away in 12 situations, 10–20 m away in 22 situations, and >20 meters away in eight situations. Results revealed that referees' positioning that resulted in the highest rate of correct decisions were when the distance were under 10 meters (83% correct decisions), good angle (88%), and good insight (86%). In contrast, referees were poorly positioned in terms of angle and/or insight in nine of the 15 erroneous decisions made. Although the present study was a preliminary qualitative investigation containing a limited number of potential penalty situations, the findings indicated that soccer referees are more likely to produce a correct decision in potential penalty situations when the distance to the situation is under 10 meters, when the insight to the situation is good and the angle to the incident is good. In contrast, the match referees generally had a poor starting position to assess the penalty situations where they landed on a wrong decision. While previous studies have been somewhat inconsistent in indicating optimal referee positioning in soccer, the present study highlights the potential value of a more qualitative approach to understand referees' positioning and subsequent decision-making accuracy.

## Introduction

Soccer referees move freely on the pitch to be appropriately positioned when enforcing the Laws of the Game (International Football Association Board IFAB, 2019). By analyzing 31 matches in the EURO 2000 finals, Helsen and Bultynck (2004) found that soccer referees typically made 137 observable decisions out of approximately 200 observable and non-observable decisions during a match. At elite levels, it is expected that referees should be in control of the game, make impartial decisions, and perform without making erroneous decisions (Samuel, 2015; Samuel et al., 2018, 2020). However, referees can and do make errors (Mascarenhas et al., 2009) that may influence the match result. Indeed, the error percentage of top-class referees was found to be approximately 14% (Mallo et al., 2012). To increase the fairness of soccer matches, it should be interesting to investigate factors that make correct decisions by referees more likely, and erroneous decisions less likely.

As the probability to score on a penalty kick in soccer is about 80% (Bar-Eli et al., 2007), potential penalty decisions are arguably some of the most important decisions made by soccer referees. The importance of penalty kicks is underpinned by the fact that the average number of goals in professional soccer is about 2.5–2.7 (Bar-Eli et al., 2007; Premier League, 2019); therefore, a decision regarding whether to award a penalty or not has a high chance of influencing the outcome of the game. One may argue that such decisions should be easy to make because the rules and guidelines regarding penalties are quite clear: “a penalty kick is awarded if a player commits a direct free kick offense inside their penalty area or off the field as part of play as outlined in Laws 12 and 13” (International Football Association Board IFAB, 2019, p. 123). Further, soccer referees must document their knowledge of the regulations and that they meet the physical requirements set for speed and endurance (UEFA, 2018).

Consequently, managing the physical requirements is a prerequisite for a referee to be adequately correct positioned in the different situations that they must consider to be able to make correct decisions (Helsen and Bultynck, 2004; Mascarenhas et al., 2006; Slack et al., 2013; Weston, 2015; Aragãoe Pina et al., 2019; Joo and Jee, 2019). In terms of distance, the Football Association UK (FA) standard says that a referee should never be more than 20 m away from the playing situation (Football Association (FA) UK, 2017) because a greater distance will increase the risk of missing important information that should be used as the basis for making a correct decision. Indeed, when investigating stress in soccer referees, Voight (2009) found that referees experience that both being misplaced when making important decisions, and making erroneous decisions in potentially match-decisive situations (e.g., penalty situations) are some of their key sources of stress. While Plessner and Betsch (2001) have argued that referees base their decisions primarily on intuition Samuel et al. (2020) have recently suggested a sequential decision-making model for understanding referees' decisions. They argue that referees' decision-making sequence consists of a series of decisions (e.g., where to run, what to anticipate, what to call), beginning with visual and attention selection and ending with evaluation of potential actions. Further, the model highlights that the decision-making sequence is influenced by multiple factors (e.g., experience, knowledge of law criteria, and referee‘s mental state), and that referees must encode relevant environmental cues to make informed decisions. Indeed, an analysis of various experts' opinions has highlighted the complex role of soccer referees, as Aragãoe Pina et al. (2019) found football referee excellence to be shaped by individual preparation, game preparation and game management. However, many situations will have both cues that indicate that the referee should award a penalty (i.e., a harsh tackle) and cues that indicate that the referee should not award a penalty (i.e., the tackler hitting the ball first). Williams et al. (1999) claim that skilled referees should know how to keep their attention on numerous stimuli and be able to differentiate between essential and less-important cues. However, when referees make important decisions with limited time, under pressure, and often with limited relevant input, it can be difficult to evaluate these cues appropriately (Wolfson and Neave, 2007; Plessner et al., 2009). Referees should therefore aim to be positioned in a way that allows them to obtain relevant cues for making correct decisions.

Hence, applicable physical fitness is required for referees to be able to keep up with the play and get an unobstructed view of potential foul play (Riiser et al., 2019). Joo and Jee (2019) highlighted in their study of Korean elite referees that both referees' physical fitness and positioning skills should be emphasized to reduce the number of referee errors throughout the match. However, although it is highlighted that soccer referees will benefit from being in the right place at the right time (e.g., Plessner and Betsch, 2001; Mascarenhas et al., 2002), the scientific evidences regarding the relationship between decision-making accuracy and referee positioning are somewhat inconclusive. For instance, de Oliveria et al. (2011) found in their study of the Brazilian soccer referees' performances that there was no association between correct calls and the referee's distance from the play. In contrast, Mallo et al. (2012) found that the distance from the referee to the event itself affected the quality of the referee's decision, with excessive distance increasing the danger of missing out on important information, while being too close to the incident increased the risk of losing track of the situation. Specifically, Mallo et al. (2012) demonstrated that an appropriate distance (11–15 m) for referees in the central zone of the playing field gave the lowest error rate in referees' decision-making, whereas the risk of incurring errors increased when referees were more distant from foul play situations. Further, Hossner et al. (2019) analyzed both distance and angle of the match referees' position relative to foul-play match infringements in all 64 matches of the 2014 FIFA World Cup. They found that referees error rates were highest when the distance to the incident were between 10 and 15 m for whistle errors and 0–5 m for non-whistle errors. IFAB (International Football Association Board IFAB, 2019) generally recommend that viewing angle of 90 degrees to the situation (i.e., from the referee's perspective, a player is attacked by the offender perpendicularly from either the left or the right side) is appropriate to gain optimal insight. However, by calculating referees' viewing angles (between 0 and 180°), Hossner et al. (2019) identified no significant effect for viewing angles on decision-making accuracy. Nevertheless, as noted by Samuel et al. (2020), when officiating soccer, the referees must use their expertise to be in the position that allows them to make a correct decision (International Football Association Board IFAB, 2019). For instance, what can be appropriate angle for detecting a tackle may be different than for detecting a handball. As an appropriate angle to the situation may vary depending on situational factors (e.g., type of incident), a more qualitative approach to analyzing referees positioning is warranted.

In summary, referees positioning skills appear to be relevant when aiming to increase accurate decision-making (Mallo et al., 2012; Football Association (FA) UK, 2017), although the empirical evidence is somewhat inconsistent (see, Hossner et al., 2019). As some of the most crucial decisions in soccer concern whether to award a penalty because of the high probability of scoring on a penalty kick (≈ 80%; Bar-Eli et al., 2007), it can be considered interesting to investigate factors that may increase the likelihood of making a correct decision in potential penalty situations in soccer (i.e., distance, angle, and insight). Thus, the aim of this study was to conduct a preliminary investigation of elite referees' positioning in the field when making a correct or an erroneous decision in penalty situations.

## Methods

### Participants

The present study used an expert panel (EP) consisting of two referees with relevant academic background to analyze referees positioning when making correct or erroneous decisions regarding penalties. The two referees in the EP were licensed and active referees and had experience at professional level in Norway. In addition, both referees had relevant academic background (i.e., degree in sport science), with one referee holding a relevant PhD. After being informed about the purpose of the study and agreeing to participate, the EP were asked to qualitatively analyze referees positioning in selected video clips by using recommended technical refereeing criteria and guidelines (i.e., the referee's distance from, angle to, and insight into the penalty situations) set by IFAB (International Football Association Board IFAB, 2019).

### Procedures

The Norwegian Social Science Data Services (NSD) provided ethical approval of the study, and the procedures were in accordance with the ethical standards of the relevant University. The EP was asked to qualitatively analyze the match referees' positions in 42 potential penalty situations. Selection of situations were based on previous research, where Erikstad and Johansen (2020) used four elite Norwegian r eferees to assess all potential penalty situations from one season of the Norwegian premier league (NPL). Specifically, Erikstad and Johansen (2020) identified 98 potential penalty situations by examining two independent objective match reports from all matches from one NPL season (*N* = 240 matches). Video clips from the potential penalty situations were collected and edited using the Camtasia Studio (Tech Smith) software to present the situations in accordance with the video assistant referee system (VAR; FIFA, 2018). Like Plessner and Betsch (2001), the clips were stopped before it was possible to identify the decision made by the match referee. Further, they muted the sound, the time and result were hidden, and the situations were shown from different angles in both fast and slow motion, as well as with zoom. After viewing a situation, the four referees were told to put a mark in the box(es) that matched their judgment of the situation (e.g., no foul, free kick to defensive team, penalty, yellow card, red card). While Erikstad and Johansen (2020) used the 98 potential penalty situations to determine potential referee biases, the present study re-analyzed video clips of the situations in which all four referees in Erikstad and Johansen's study considered that the match referee made a correct decision (*N* = 28) or an erroneous decision (*N* = 14). The 14 situations where the match referee made an erroneous decision are previous studied (see Johansen and Erikstad, 2018) but are included in present paper for comparison of the analysis of the situations where the match referee made a correct decision.

### Analysis

A video-based analysis of 42 selected penalty situations was conducted with the background of national and international officiating technical variables related to the referee's placement and movement (Football Association (FA) UK, 2017; UEFA, 2018). Based on findings from previous studies (e.g., Mallo et al., 2012), the distance between the referee and the penalty situation was estimated from the referee's position in relation to various markings on the field and further thematically categorized as short (<10 m), average (10–20 m), or long (>20 m). Further, the referees' guidelines (International Football Association Board IFAB, 2019) highlight that referees must use their expertise to be in the position that allows them to make a correct decision. The EP further qualitatively analyzed the referees' position in terms of angle and insight into the various situations. While insight was defined as whether one or more players blocked the referee's view, the expert panel determined the quality of the angle into the situation based on their experience and expert knowledge, as appropriate angle to the situation may vary depending on situational factors (e.g., type of incident). Based on their evaluation, good angle was obtained when the referee were in a position where they clearly could see a gap between the involved players' bodies and the relevant actions, allowing the referee to observe potential contact between the defender and attacker. Although good angle depends on situational factors, the EP noted that a good angle most often is obtained when the viewing angle is between 45 and 135° to the infringement (see Hüttermann et al., 2017; Hossner et al., 2019 for more information about how viewing angles can be calculated). Poor angle represented situations where details of the situation was hidden due to no gap between players and the related action. Poor angle typically occurred when the attacker was between the defender and referee or the defender was between the attacker and referee (i.e., close to either 0 or 180°). Average angle represented situations where details of the situations were only partly visible due to small gap between involved players' bodies. The results were thematically divided into the categories good, average, or poor. While the EP were shown the situations from three or more angles and in normal and slow-motion, they were like Erikstad and Johansen (2020) told to skip that situation if they felt the video did not expose the situation sufficiently. However, the EP reported that the quality of the clips was adequately presented for all 42 situations.

The organization and categorization in the analysis of the various video clips was carried out in accordance with general guidelines for analyzing qualitative research inspired by a thematic analysis (Braun and Clarke, 2006). An overall aim in thematic analysis is that the themes operationalized in the different categories are strongly linked to the data (Braun et al., 2016), which were the various video clips in this case. The analysis of the video clips was performed by two independent persons, as recommended for increasing validity in studies using subjective analysis (Thomas et al., 2015). Both were authorized soccer referees with professional experience in Norway, and one had relevant research experience. The correspondence between the two experts in the thematic analysis of the different situations was 98%, which is very high (Pearce et al., 2010). The one situation containing disagreement were subsequently discussed by the EP, and a mutual analysis was produced.

Due to the limited number of situations, results are descriptive comparisons of the EP‘s evaluations of distance, angle, and insight in the situations where the actual match referee made a correct and erroneous decision.

## Results

Overall, the EP evaluated 42 situations, of which the actual match referee made a correct decision in 28 situations (67%) and the remaining 14 situations were incorrect (33%). Regarding distance, the results revealed that the referee was <10 meters away in 12 situations (29%), 10–20 m away in 22 situations (52%), and >20 meters away in eight situations (19%). For angle, the EP considered that the match referees had a good angle in 25 situations (60%), average angle in seven situations (17%), and poor angle in 10 situations (24%). Finally, regarding insight, the EP considered the match referee to have a good insight in 21 situations (50%), average insight in 10 situations (24%), and poor insight in 11 situations (26%). Of the 42 situations, 29 (69%) were related to tripping infringements, and the remaining situations were related to pushing (eight situations; 19%), handball (three situations; 7%) and shirt holding (two situations; 5%) infringements. Distance, angle, and insight in relation to type of infringements and correct and erroneous decisions are presented below (see Tables 1, 2).

**Table 1:** Characteristics of distance, angle, and insight in penalty situations where the match referee had ruled correctly.

**Video clip**	**Distance**	**Angle**	**Insight**	**Decision**	**Infringement**
4	Short	Good	Good	Penalty	Leg tripping
8	Short	Good	Good	Penalty	Leg tripping
13	Short	Good	Good	Penalty	Leg tripping
15	Average	Good	Average	Penalty	Leg tripping
17	Short	Good	Good	Penalty	Leg tripping
24	Average	Poor	Average	Penalty	Leg tripping
25	Average	Good	Average	Penalty	Leg tripping
28	Average	Good	Good	Penalty	Leg tripping
29	Average	Good	Average	Penalty	Leg tripping
30	Average	Poor	Average	Penalty	Leg tripping
31	Short	Good	Good	Penalty	Leg tripping
34	Long	Poor	Poor	Penalty	Leg tripping
35	Average	Good	Good	Penalty	Holding
36	Long	Poor	Poor	Penalty	Leg tripping
1	Average	Good	Good	No penalty	Pushing
2	Average	Good	Good	No penalty	Pushing
11	Short	Good	Good	No penalty	Leg tripping
15	Short	Good	Good	No penalty	Handball
16	Short	Good	Good	No penalty	Leg tripping
18	Long	Good	Good	No penalty	Leg tripping
21	Short	Good	Good	No penalty	Leg tripping
22	Long	Good	Average	No penalty	Leg tripping
23	Average	Good	Good	No penalty	Pushing
26	Average	Average	Average	No penalty	Holding
27	Short	Average	Poor	No penalty	Leg tripping
33	Average	Good	Good	No penalty	Pushing
38	Average	Good	Good	No penalty	Pushing
42	Average	Good	Good	No penalty	Handball

**Table 2:** Characteristics of distance, angle, and insight in penalty situations where the match referee had ruled incorrectly.

**Video clip**	**Distance**	**Angle**	**Insight**	**Decision**	**Infringement**
3	Average	Poor	Average	No penalty	Pushing
6	Average	Average	Average	No penalty	Pushing
7	Short	Average	Average	No penalty	Leg tripping
9	Long	Poor	Poor	No penalty	Leg tripping
10	Long	Poor	Poor	No penalty	Leg tripping
12	Average	Good	Good	No penalty	Leg tripping
14	Long	Poor	Poor	No penalty	Leg tripping
32	Short	Good	Poor	No Penalty	Leg tripping
37	Average	Average	Poor	No penalty	Leg tripping
39	Long	Poor	Poor	No penalty	Leg tripping
41	Average	Average	Good	No penalty	Leg tripping
5	Average	Poor	Poor	Penalty	Leg tripping
20	Average	Good	Good	Penalty	Handball
40	Average	Average	Poor	Penalty	Pushing

Regarding distance, the match referee was positioned within 10 m from the incident in 12 situations, of which 10 (83%) were evaluated correctly (see Table 1). For distances between 10 and 20 meters (*N* = 22), referees made a correct decision in 14 situations (64%) and incorrect in eight situations (36%). When the match referee was positioned over 20 meters away from the situations (*N* = 8), four of eight situations (50%) were correctly refereed. Regarding angle, the referee made a correct decision in 22 of the 25 situations (88%) where the EP evaluated the positioning as good. When the match referee had an average angle to the situation (*N* = 7), the match referee was correct in two situations (29%).When having a poor angle (*N* = 10), referees made a correct decision in four situations (40%). Finally, regarding insight, the match referees made a correct decision in 18 of the 21 situations (86%) when the EP evaluated that their position allowed them to have good insight into the situation. For average positioning regarding insight, the match referee made a correct decision in seven of 10 situations (70%). When being poorly positioned in terms of insight, the match referee made a correct decision in three of 11 situations (27%). The positioning that were associated with the highest rate of correct decisions were thereby distances of under 10 meters (83% correct decisions), good angle (88%), and good insight (86%). Furthermore, no erroneous decisions were made in the nine situations where referees were under 10 m away from the situation, and where the angle and insight were good. In contrast, referees made an erroneous decision in nine of the 14 situations (64%) where either the angle or the insight were poor (See Table 2).

## Discussion

The present study was a preliminary investigation of referees' positioning in potential penalty situations where they made a correct decision and where they made an erroneous decision. Based on a qualitative analysis of two expert referees, the results revealed that the highest rate of correct decisions by the match referees occurred when the distance to the incident were under 10 m (83% correct decisions), and when the EP determined that the match referee had good angle (88%) and good insight (86%) into the situation. Erroneous decisions were more likely to occur with extended distance to the situation (36% incorrect with distances 10–20 m, and 50% with distances exceeding 20 meters). The referee made an erroneous decision in nine of the 14 situations (64%) where the EP considered that either the angle or the insight were poor. The results may indicate that appropriate positioning (i.e., short distance, good angle and good insight to the situation) allows referees to make decisions based on solid cues, and that inappropriate positioning may lead to limited visual input and increased risk of erroneous decisions.

The findings of current study indicated that good quality of referees' positioning appear to increase the likelihood of making a correct decision in penalty situations. The placement of the referee at an optimal angle and with good insight into the match play appeared to be relevant for a correct decision on whether to award or not award a penalty. These findings are in correspondence with the refereeing criteria and practical guidelines set IFAB (International Football Association Board IFAB, 2019) and seemed to provide the referee with views of the situations that enable them to attend to important and essential cues that indicate whether a penalty kick should be awarded or not. Further, the present findings are in line with the arguments provided by Samuel et al. (2020), which note that appropriate positioning by soccer referees will influence the likelihood of making correct decisions. Indeed, as Williams et al. (1999) suggested, skilled referees should know how to keep their attention on numerous stimuli and be able to differentiate between essential and less-important cues. The present findings may thereby indicate that short distance and good angle and insight allows referees to base their decisions on solid cues. This may be particularly important to detect tripping offenses, which were the most typical infringement in the present study (69% of the situations). Indeed, being close and with a clear view and angle may be crucial to identify a contact between players legs, and thus making an informed decision.

While the findings of present study have indicated that appropriate positioning of the match referee appear beneficial for making a correct decision in a potential penalty situation, appropriate positioning is not always the case. Regarding distance, the present study identified that the referee had a long distance (> 20 m) in eight situations, of which four was incorrectly refereed. Previous studies have been somewhat inconsistent in identifying appropriate distance to foul situations (e.g., de Oliveira et al., 2011; Mallo et al., 2012). However, of the misjudged 14 situations, the referee had extended distance in four situations, poor insight in eight situations and poor angle in six situations. The findings may thereby indicate that appropriate angle and insight into the situation is important for identifying a potential penalty kick. This appear particularly relevant to avoid making a non-whistling error, as 11 of 14 misjudged penalty situations where penalty kicks were not given. This is in line with previous evidence that referees error rates are lower in situations where they whistle compared to situations where they avoid whistling (Hossner et al., 2019). Furthermore, the findings of present study demonstrated that when the match referees made a correct decision by not awarding a penalty kick both the angel and insight to situation were good or average in all (14) but one situation (see Table 1). In the situations where a correct decision was to award a penalty kick the angle and insight were good or average in 10 of the 14 situations (see Table 1). The results thereby indicate that good angle and insight for the match referee may contribute to errorless decision-making in potential penalty situations. However, the findings regarding viewing angles differs from Hossner et al. (2019), which identified no significant effect for calculated viewing angles on decision-making accuracy. In combination, the findings may thereby indicate that appropriate viewing angles depends on situational factors, but generally is obtained when the referee is able to see a gap between the involved players' bodies and the relevant actions (i.e., most often between 45 and 135° to the infringement).

Potential penalty situations are potentially match-decisive decisions (Bar-Eli et al., 2007). The misplacement of the referees of present study may have forced them to make these important decisions in a limited time and it might be possible that their judgement of the importance of the cues has been affected. The referees' mispositioning in the field of play might have created uncertainty, and Johansen and Erikstad (2018) used error management theory (Haselton and Nettle, 2006), which says that decisions made under uncertainty will be biased toward the least costly error, to explain their findings. Specifically, it may be that a wrongful given penalty is perceived as a bigger error than a wrong dismissed penalty. However, it is important to emphasize that the soccer referee is human and makes quick decisions based on a subjective assessment of various play situations (Poolton et al., 2011). Even if he or she can move freely on the playing field to access the best possible distance, angle, and insight, the referee does not always have optimal insight into a situation and must decide based on their intuition and the environmental cues obtained (Plessner et al., 2009; Samuel et al., 2020). Nevertheless, as research has indicated that referees may be influenced by social pressure (Sutter and Kocher, 2004; Erikstad and Johansen, 2020), and that errors thereby not necessarily are equally distributed across teams, appropriate positioning in penalty situations as demonstrated in our study may also contribute to reduce the risk of (unintentionally) biased decisions.

### Strengths and Limitations

Selection of video clips was based on previous empirical work where referees unanimously agreeing that the referee had either made a correct or an erroneous decision (see Erikstad and Johansen, 2020). Therefore, there is a basis for claiming that these situations represent situations where the judgments are most likely to have been correct. Further, the qualitative video-based analysis was performed by two persons with experience from refereeing at professional level, and relevant academic background. Weston (2015) claims that that the use of such EP is a suitable method when examining the degree of accuracy of a judicial decision. The correspondence between the two independent experts in their characteristics of distance, angle, and insight into the different situations was 98%, which is considered very high (Pearce et al., 2010).

Nevertheless, the study had limitations that must be considered. First, the study included a limited number of situations, and statistical comparisons were therefore not considered appropriate. Consequently, the present study is considered a preliminary investigation, and the findings may not be generalized. Further, while the EP could refrain from evaluate a situation if they felt the video clip did not provide sufficient information, the lack of complete insight into the video clips and the ability to assess what the referee could have seen makes the categorizations done somewhat uncertain. The categorizations should therefore be viewed as indications and future research could include eye-tracking to better understand referees' visual input and subsequent decisions. Also, while the experimental design allowed the EP to categorize situations based on their experience and expertise, the lack of objective measures (e.g., appropriate angle) is highlighted as a potential limitation. Moreover, any communication between referees and assistant referees was unknown. Messages may have been exchanged between the referees on the internal communication network and might have influenced the decisions that were made. Knowledge of the contents of the internal communication between the referees would have provided valuable information about the match referee's possible uncertainty and doubts in the various penalty situations.

## Conclusion and Practical Implications

In conclusion, the present preliminary investigation of referees positioning in potential penalty situations indicates that referees are most likely to make a correct decision when the distance to the incident are under 10 m, and when the angle and insight to the situation is good. Thus, referees' positioning skills may therefore be highlighted in both referees‘ training programs, and when evaluating their performances. Indeed, Samuel et al. (2020) notes that the first decision for the referee to make is where to run on the field, and often how fast he or she should run. Based on the findings of the present preliminary investigation, referees may benefit from being aware that most potential penalty incidents relates to tripping infringements, thus highlighting the need have a clear view of the players legs through short distance and clear view and angle (i.e., a viewing angle that allows the referee see a gap between the involved players' bodies and the relevant actions). The referee's optimal placement and subsequent correct decision-making in various penalty situations uncovered in this study also indicates the importance of referees being physically and mentally prepared. Specifically, as the highest rate of correct decisions was achieved when being placed <10 m from the incident, the present study adds to the literature highlighting the importance of referees physical capabilities (Joo and Jee, 2019; Riiser et al., 2019). However, physical fitness should be combined with referees positioning skills, as the present findings indicates that good angle and insight is beneficial to make a correct decision. Referees may therefore benefit from including positioning skills in their training, for instance through observations and video analysis. It would also be interesting to expose the match referees to these situations to investigate what they perceived and thought at the time, and how they consider and assess these situations in retrospect. Such knowledge may lead to the use of individual video clips and to personalize decision-making training of referees. Especially fruitful may such training be if the video game clips, as suggested by Raab et al. (2020), include the context of the game to show sequences of decisions including the sequentially previously made choices (e.g., the referees' preferred positioning in potential penalty situations for making a decision).

## Data Availability Statement

The original contributions presented in the study are included in the article/supplementary materials, further inquiries can be directed to the corresponding author/s.

## Ethics Statement

The studies involving human participants were reviewed and approved by Research Ethics Committee, Faculty of Health and Sports Sciences, University of Agder. The patients/participants provided their written informed consent to participate in this study.

## Author Contributions

BJ developed the study rationale and design, interpreted the data, drafted, and revised the article. ME contributed to the development of the study rationale and design, collected, interpretation of data, drafted, and revised the article.

## Conflict of Interest

The authors declare that the research was conducted in the absence of any commercial or financial relationships that could be construed as a potential conflict of interest.
